# Neuroanatomical and neurophysiological evidence of pulmonary nociceptor and carotid chemoreceptor convergence in the nucleus tractus solitarius and nucleus ambiguus

**DOI:** 10.1152/jn.00125.2022

**Published:** 2022-04-20

**Authors:** Jekaterina Zyuzin, Nicholas Jendzjowsky

**Affiliations:** Respiratory and Critical Care Physiology and Medicine, and Neurotherapeutics, The Lundquist Institute for Biomedical Innovation, Harbor UCLA Medical Center, Torrance, California

**Keywords:** carotid chemoreceptor, nucleus ambiguus, nucleus tractus solitarius, pulmonary nociceptor, sensory convergence

## Abstract

Pulmonary vagal nociceptors defend the airways. Cardiopulmonary vagal nociceptors synapse in the nucleus tractus solitarius (NTS). Evidence has demonstrated the convergence of cardiopulmonary nociceptors with afferents from carotid chemoreceptors. Whether sensory convergence occurs in motor nuclei and how sensory convergence affects reflexive efferent motor output directed toward the airways are critical knowledge gaps. Here, we show that distinct tracer injection into the pulmonary nociceptors and carotid chemoreceptors leads to co-labeled neurons in the nucleus tractus solitarius and nucleus ambiguus. Precise simultaneous stimulation delivered to pulmonary nociceptors and carotid chemoreceptors doubled efferent vagal output, enhanced phrenic pause, and subsequently augmented phrenic motor activity. These results suggest that multiple afferents are involved in protecting the airways and concurrent stimulation enhances airway defensive reflex output.

**NEW & NOTEWORTHY** Sensory afferents have been shown to converge onto nucleus tractus solitarius primary neurons. Here, we show sensory convergence of two distinct sets of sensory afferents in motor nuclei of the nucleus ambiguus, which results in augmentation of airway defense motor output.

## Graphical abstract

**Figure:**
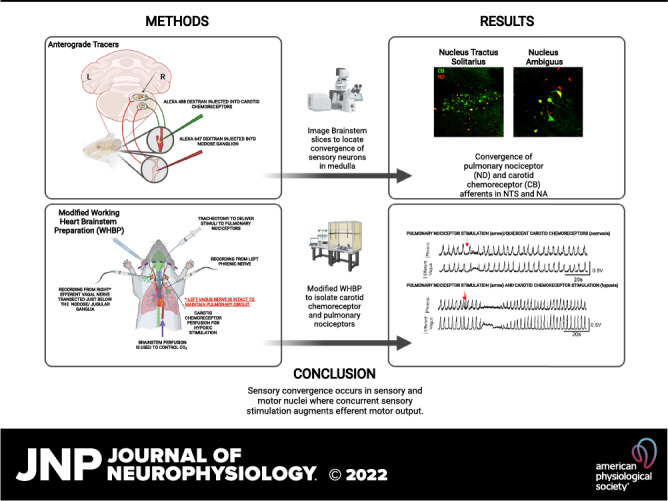

## INTRODUCTION

Pulmonary nociceptors, housed in the vagus, sense and respond to mechanical distension (lung stretch, Aδ fibers) and noxious stimuli/immune processes (lung irritants/cytokines via c-fibers/nociceptors) leading to efferent vagal activity that evokes airway defensive reflexes (1). Pulmonary nociceptors synapse primarily in the nucleus tractus solitarius (NTS), the main medullary sensory hub. Postsynaptic NTS neurons then project onto the Bötzinger and PreBötzinger complex and nucleus ambiguus (NA); all of which go on to activate the motor end of the reflex arc (2–4). Carotid chemoreceptors also synapse in the NTS (5). Previous data from in vivo and in vitro single NTS neuron recordings (6–9) showed that carotid chemoreceptor and vagal nociceptor projections synapse onto shared NTS neurons. However, previous data could not precisely control stimulus delivery to each sensory compartment nor attempted concurrent stimulation of multiple afferents. Further, these investigations did not characterize how stimulation of multiple afferent sets would alter resulting motor output with a key role of protecting the airways.

Teleologically, dual sensory activation of vagal pulmonary nociceptors and carotid chemoreceptors could signal an enhanced threat to the airways and cardiorespiratory system, likely requiring increased protection. Therefore, it is possible that activation of both pulmonary nociceptors and carotid chemoreceptors could augment motor output to the upper airways and suppress initial phrenic activity to evoke airway defensive reflexes due to sensory convergence in sensory and motor nuclei. To date, whether sensory convergence extends beyond the NTS and what significance it has on the efferent end of the airway defense reflex arc has not been tested.

We tested the hypothesis that simultaneous co-stimulation of carotid chemoreceptors and pulmonary nociceptors would enhance airway defensive reflex output using a modified variation of the working heart brainstem preparation (WHBP) (10) named the dual perfused preparation (11). To investigate specific pulmonary nociceptor and carotid chemoreceptor co-stimulation, our experimental preparation retained an intact pulmonary circuit, removed in previous models (11–13) and, precisely controlled delivery of sensory stimulation while recording from the efferent vagus and phrenic nerves. We validated the electrophysiological outcomes with tracer injection into both afferent ganglia and identified a multitude of shared neurons in brainstem nuclei. The data presented herein support our hypothesis that dual cardiorespiratory afferent stimulation augments airway defensive reflex motor output, supported by a high degree of synaptic convergence in the NTS and NA.

## METHODS

### Animals

Experimental procedures were approved by The Lundquist Institute for Biomedical Innovation at Harbor UCLA Institutional Animal Care and Use Committee (Protocol No. 32183) and performed in accordance with guidelines set forth by the National Institutes of Health Guide for the Care and Use of Laboratory Animals (8th edition, 2011). Male Sprague-Dawley rats (SD, p21–28, 40–80 g, Charles River, CA) were housed in pairs in a 12-h light/dark cycle with water and chow freely available until surgical procedures (*Anterograde Tracing and Immunohistochemistry*).

### Modified WHBP

Sprague-Dawley rats (80–150 g) were deeply anesthetized in isoflurane (5%, balance O_2_) and prepared as described previously (11, 14) with modifications. Once deeply anesthetized, as assessed by near absent respiratory movement, lack of paw-pinch and corneal reflex, rats were cooled in ≤4°C physiological saline (ACSF, 115 mM NaCl, 24 mM NaHCO_3_, 4 mM KCl, 2 mM CaCl_2_, 1 mM MgSO_4_, 1.25 mM NaH_2_PO_4_, 10 mM glucose, and 12 mM sucrose, equilibrated with 95% O_2_-5% CO_2_, pH 7.4), decerebrated at midcollicular level, transected above the renal arteries, and skinned. The descending aorta was cannulated with a double-lumen catheter; one lumen was connected to a peristaltic pump (Gilson Minipuls 3, Middleton, WI) to perfuse with ACSF. The other lumen was attached to a pressure transducer and used to monitor perfusion pressure (AD Instruments, Colorado Springs, CO) maintained at ∼90 mmHg (12–16 mL·min^−1^). The common carotid arteries were separately perfused at 18.5 mL·min^−1^ to maintain a pressure of ∼90 mmHg. The central perfusate was equilibrated with 40 Torr Pco_2_ in O_2_, and the peripheral perfusate was equilibrated with 35 Torr Pco_2_ and 100 Torr Po_2_ in N_2_ with a gas mixer (GSM-3 CWE Inc., Ardmore, PA) at 31 ± 1°C. Note, the decerebration transects the circle of Willis, which prevents any mixing of perfusates. The phrenic nerve and right vagus nerve were transected just below the nodose/jugular complex and attached to suction electrodes. The left vagus was kept intact to maintain the pulmonary afferent circuit to the brainstem. A tracheotomy allowed perfusion of pulmonary stimulants directed to the pulmonary afferents. This allowed discrete delivery of stimuli to carotid chemoreceptors and pulmonary nociceptors as other receptors such as the aortic chemoreceptors and dorsal root ganglia were perfused by the central brainstem perfusate which was tightly controlled. Raw neurograms were amplified (10,000× phrenic, 20,000× vagus; Differential AC Amplifier Model 1700, A-M Systems Inc., Carlsborg, WA), filtered (low cut-off, 300 Hz, high cut-off, 5 kHz), rectified and integrated, and computer archived using Powerlab16/35 and Labchart Pro software (AD Instruments, Colorado Springs, CO) at 5 kHz.

Typically, carotid body excitation is increased when brainstem chemoreceptors are suppressed with hypocapnic perfusate (13). To test the full co-stimulation of vagal defensive augmentation we conducted both brainstem normocapnic/carotid body hypoxic and brainstem hypocapnic/carotid body hypoxic conditions. Our carotid chemoreceptor/brainstem stimulation parameters were: *1*) brainstem normocapnia (40 Torr CO_2_), carotid body normoxia (100 Torr O_2_, NcNx); *2*) brainstem normocapnia (40 Torr CO_2_), carotid body hypoxia (40 Torr O_2_, NcHx); and *3*) brainstem hypocapnia (<15 Torr CO_2_), carotid body hypoxia (40 Torr O_2_, HcHx). Pulmonary nociceptors were stimulated with adenosine triphosphate (ATP, Millipore Sigma Cat. No. A3377, 0.05 mg in 50-μL PBS, *n* = 8 individual experiments) or fungal protease XIII (Millipore Sigma, Cat. No. P2143 0.375 mg in 50-μL PBS *n* = 6 individual experiments).

Phrenic and efferent vagal activity was analyzed from raw traces using the built-in spike analyzer in LabChart (AD Instruments). The magnitude (μV) of both neurograms were multiplied by respective frequencies to calculate total activity (normalized units). Baseline was considered the 60 s before pulmonary nociceptor stimulation. The response consisted of the 60 s encompassing pulmonary nociceptor stimulation. The percentage change in response to pulmonary nociceptor stimulation was calculated to compare between Nc/Nx, Nc/Hx and Hc/Hx conditions to account for changes in baseline. The delay in phrenic firing was calculated as the time between distinctive bursts and calculated as a percent change from Nc/Nx.

### Anterograde Tracing and Immunohistochemistry

Sprague-Dawley rats (*n* = 4, 80–100 g) were anesthetized with ketamine/xylazine (90 mL^.^kg^−1^/10 mL^.^kg^−1^, ip). Once a surgical plane of anesthesia was attained as assessed by lack of paw-withdrawal and corneal blink reflex, the neck was shaved and sterilized with 3× betadine and 70% alcohol. The carotid chemoreceptors and the nodose ganglion were exposed and 3 μL of 10% Alexa 488 Dextran (10,000 kDa MW, Invitrogen Cat. No. D22910, Ex/Em λ495/519- carotid chemoreceptors) and 3 μL of 10% Alexa 647 (10,000 kDa MW, Invitrogen Cat. No. D22914 Ex/Em λ650/668- nodose/jugular ganglion), dissolved in 0.1 M phosphate buffer (PB), were pressure injected into right side ganglia at a rate of 5 nL/s (Nanoject III, Drummond Scientific, Brummal, PA). Because of the close proximity of the nodose/jugular ganglion and carotid chemoreceptors, Retrobeads (Lumafluor, Naples, FL) were coinjected into carotid chemoreceptors (Green Ex/Em λ460/505) and nodose/jugular ganglion (Red Ex/Em λ530/590) to confirm distinct and accurate tracer injection. The left side served as an internal negative control. Rats were treated postoperatively with subcutaneous fluids (3 mL, 0.9% saline) and buprenorphine (0.03 mL^.^kg^−1^ SQ, Reckitt Benckiser Pharmaceuticals, Richmond, VA). Rats recovered for 11 days in singly housed enclosures.

On the 11th day, rats were deeply anesthetized with isoflurane (5%, balance O_2_) and once a deep plane of anesthesia was attained as assessed by slowed respiratory rate and lack of blink and hind-limb withdrawal reflexes, animals were pericardially perfused with ∼400 mL Ringers solution (pH 6.9) and then switched to 4% paraformaldehyde (in 0.1 M phosphate buffer, PB). Brains, carotid chemoreceptors, and nodose/jugular ganglia were collected and fixed for 1 h and then placed in 30% sucrose (in 0.1 M PB) for >24 h at 4°C.

The region of the NTS was matched to atlas levels between −15.96 mm and −11.64 mm from bregma (15). The NA was identified lateral and ventral to the NTS and cells were counted. Positive dextran tracer-labeled cells displayed cytosolic labeling surrounding a blank nuclear region with a majority of positively labeled cells displaying axonal labeling, as has been previously described (16–18). To characterize nuclei, additional sections were stained with choline acetyltransferase (ChAT, raised in rabbit, 1:100 Millipore-Sigma Cat. No. ZRB1012) and vesicular glutamate transporter 2 (Vglut2, raised in mouse, 1:100 Abcam Cat. No. ab211869) and secondary antibodies (Dylight 405 Ex/Em λ435/460, anti-rabbit, Jackson Immuno, Cat. No. 111–475-003; Cy3 Ex/Em λ555/569, anti-mouse, Jackson Immuno, Cat. No. 115–165-146), which do not overlap with spectra from each other or anterograde tracers above. Antibodies were chosen based on Western blot validation by the manufacturer. Additionally, no primary controls were run in tandem.

Sections of medulla (40 μm) from −15.5 mm to −11.5 mm from bregma were attained and mounted onto gelatin-coated slides. Slides were coverslipped using Prolong Diamond Antifade Aqueous Mounting Medium (P36961 - Invitrogen) and kept at 4°C until imaging. Images were taken with Leica Thunder 3 D Cell Culture Imaging system or SP8 confocal imaging systems as indicated in figure legends. Cells were counted manually using LasX software.

### Data Analysis

All data are presented as means ± SD. Statistical analyses were performed with GraphPad 6.0. Electrophysiological data were compared by repeated-measures ANOVA and Holm–Sǐdak post hoc test (correcting for multiple comparisons). Proportions of co-labeled cells were assessed with χ^2^ test of proportion. Statistical significance was accepted at *P* < 0.05.

## RESULTS

**Figure 1.**
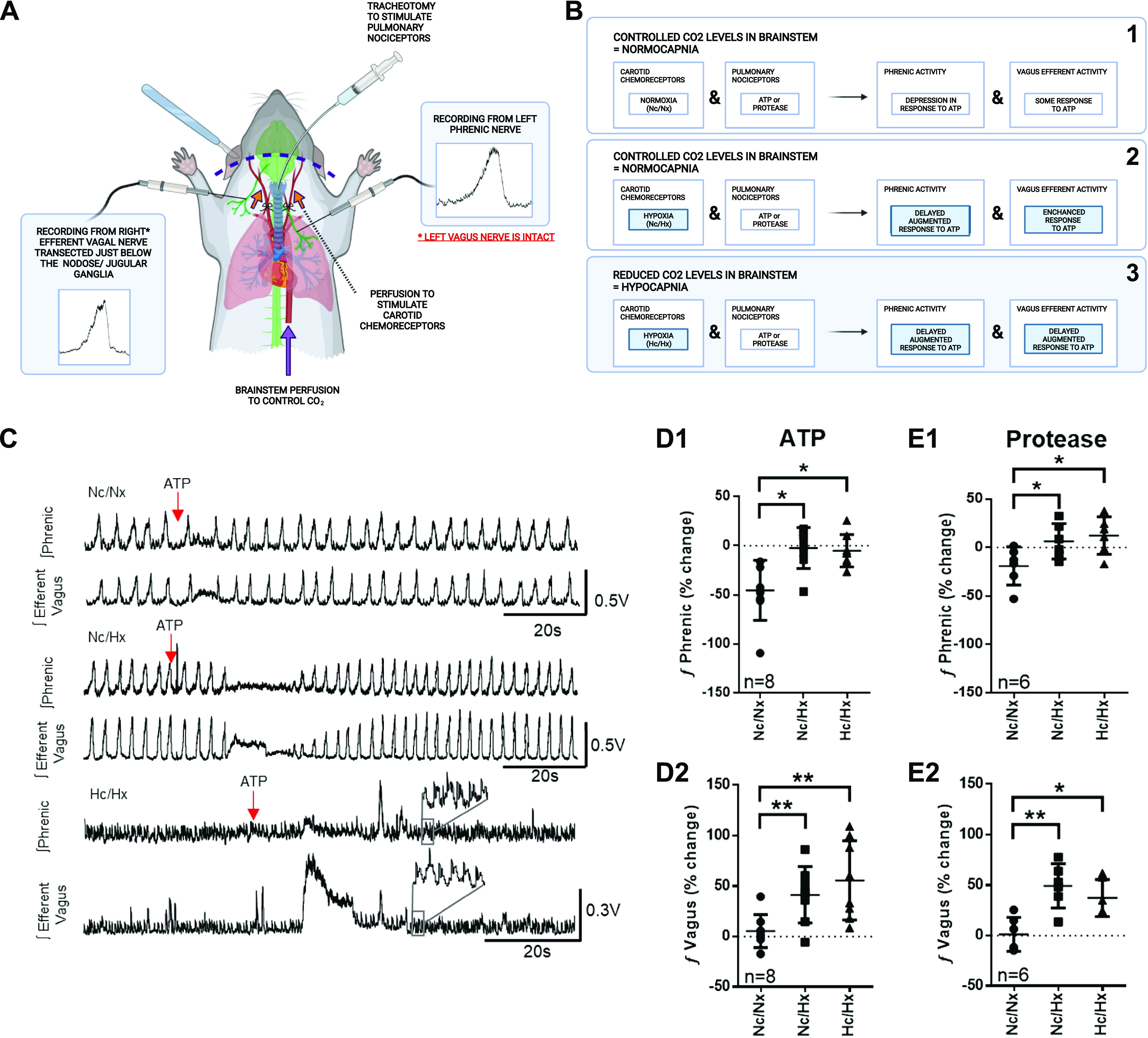
Heightened efferent vagal excitation in response to concurrent pulmonary nociceptor and carotid chemoreceptor excitation. *A*: the schematic representation demonstrating single waveforms of efferent vagus and phrenic nerve firing. The perfusions are isolated to specifically stimulate pulmonary nociceptors via tracheal injection or carotid chemoreceptors via carotid perfusion. Perfusion to the brainstem was used to control CO_2_ to reveal the full carotid body excitation. *B*: a schematic to summarize the data. *C*: representative traces of phrenic and efferent vagal nerves during concurrent pulmonary nociceptor stimulation and carotid chemoreceptor quiescence during normoxia (Nc/Nx, *top*, corresponding to *B1*), carotid body stimulation with hypoxia (Nc/Hx, *middle*, corresponding to *B2*), and brainstem hypocapnia/carotid chemoreceptor hypoxia (Hc/Hx, *bottom*, corresponding to *B3*) show that hypoxia augments vagal efferent activity in response to ATP (0.05 mg, ATP) delivered to the lungs. Nc/Nx (brainstem normoxia/normocapnia/carotid body normoxia/normocapnia); Nc/Hx (brainstem normoxia/normocapnia/carotid body hypoxia/normocapnia); Hc/Hx (brainstem normoxia/hypocapnia/carotid body hypoxia/normocapnia). *C* and *E*: % change from baseline of phrenic (*D1*) and efferent vagal (*D2*) activity in response to ATP (0.05 mg in 50-μL PBS). % Change from baseline of phrenic (*E1*) and efferent vagal (*E2*) activity in response to fungal protease (0.0375 mg in 50-μL PBS). *D1*: F_2_ = 9.15, *P* = 0.003. *D2*: F_2_ = 14.59, *P* = 0.0005. *E1*: F_2_ = 7.19, *P* = 0.016. *E2*: F_2_ = 15.81, *P* = 0.004. *D1* and *D2*: *n* = 8 separate animal experiments. Each data point is a single animal from one experiment. *D1* and *D2* were recorded simultaneously from the same animal. In *E1* and *E2*: *n* = 6 separate animal experiments. Each data point is a single animal from one experiment. *E1* and *E2* were recorded simultaneously from the same animal. Post hoc, Holm–Sidak, *P < 0.05, **P < 0.001. Image created with BioRender and published with permission.

Prior studies examining a limited number of NTS neuron recordings indicated convergence of sensory information from vagal pulmonary afferents and carotid chemoreceptors (6–9). Furthermore, supporting a role for carotid body chemoreceptors in airway defense are results of their stimulation which augments apnea, bronchoconstriction, and cough (14, 19–22). We, therefore, tested the hypothesis that sensory convergence from pulmonary nociceptors and carotid chemoreceptors in the NTS and NA coregulate vagal airway defense efferent activity. To test this, we modified the dual-perfused preparation (11–13), a variant of the WHBP. In our preparation, the lungs are in place, and the vagal afferents on the left side are intact, while we recorded from the transected right vagus just below the nodose/jugular complex. These modifications were necessary to capture defensive airway motor output and manipulate vagal and carotid pathways independently while simultaneously measuring phrenic and efferent vagal activity (Fig. 1*A*). By perfusing the brainstem, carotid chemoreceptors, and lungs separately, our experiments are the first to precisely control multiple stimulus delivery to pulmonary nociceptors, carotid chemoreceptors, and the brainstem both independently and simultaneously.

We stimulated pulmonary nociceptors with ATP or a fungal protease. This allowed us to test whether afferents related to tracheal irritation (23–25) and innate immune sensing (26, 27) would elicit similar vagal efferent activity (Fig. 1). Simultaneously, we stimulated the carotid chemoreceptors with hypoxia mimicking what would occur should lung function decline as a result of airway narrowing. During basal conditions, we saw little change or a slight reduction of phrenic motor output and an increase in vagal activity in response to both stimuli (Fig. 1, *C*–*E*). During dual carotid chemoreceptor and pulmonary nociceptor stimulation, the delay of phrenic firing (time between bursts, normalized to Nc/Nx) was lengthened during Nc/Hx [ATP (*n* = 8): 0.94 ± 0.67; protease (*n* = 6): 0.58 ± 1.35 times longer than Nc/Nx, *p* < 0.05] and Hc/Hx [ATP (*n* = 8): 2.31 ± 3.29; protease (*n* = 6): 1.64 ± 1.39 times longer than Nc/Nx, *P* < 0.05] suggestive of a greater apnea. Total phrenic activity in response to ATP and protease during Nc/Hx and Hc/Hx was augmented (Fig. 1, *D* and *E*), likely to counteract the lengthened pause. Efferent vagal activity was augmented in response to ATP or protease as seen during both Nc/Hx and Hc/Hx conditions (Fig. 1, *D* and *E*).

**Figure 2.**
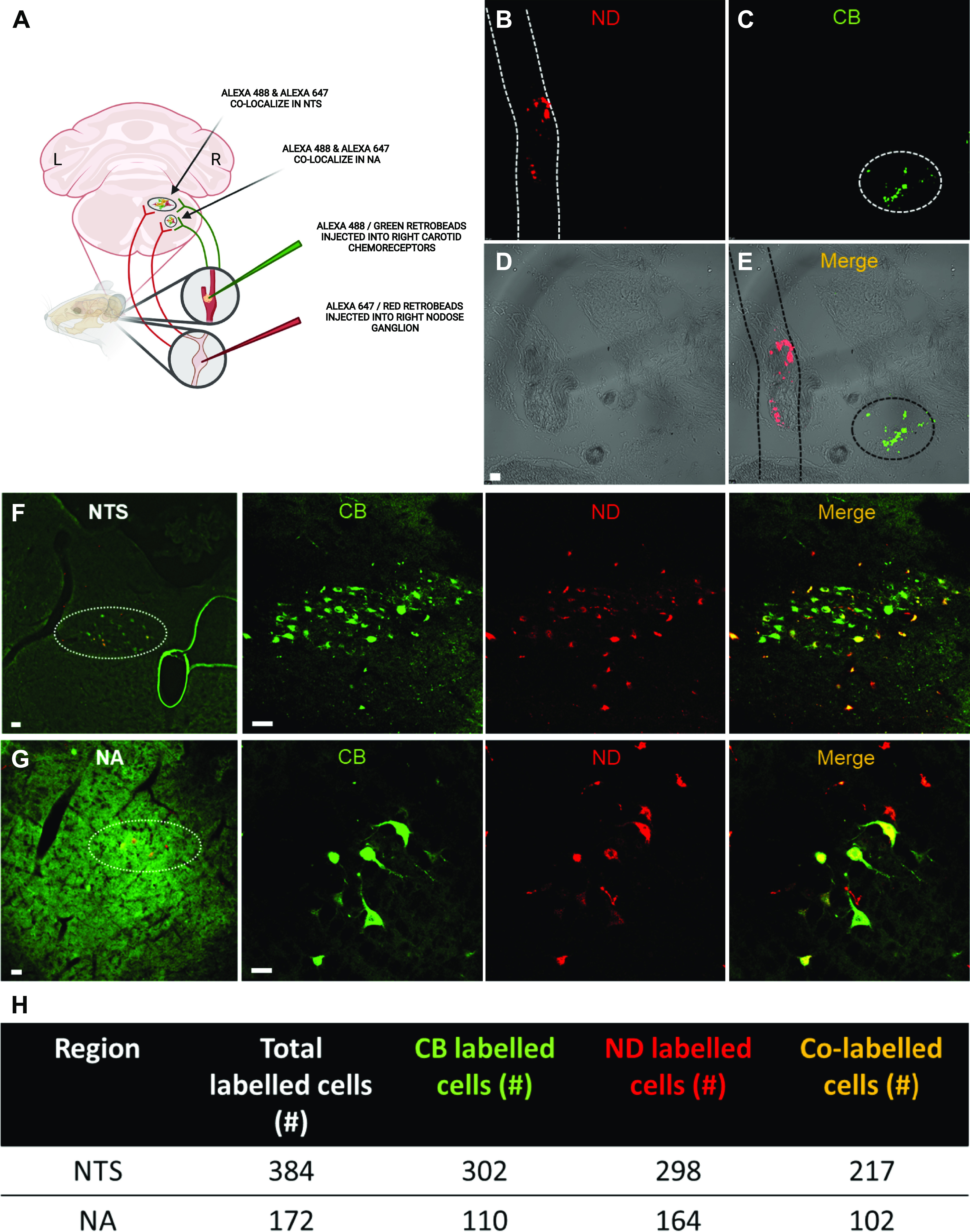
Pulmonary nociceptor and carotid chemoreceptor anterograde tracer injection demonstrates overlapping first order neurons in the nucleus tractus solitarius and nucleus ambiguus. *A*: schematic representation of discrete injection of 10,000 MW Alexa 488-conjugated Dextran injected into the right carotid chemoreceptors (CB) and 10,000 MW Alexa 647-conjugated Dextran injected into the right nodose ganglion (ND) housing the pulmonary nociceptor cell bodies. *B–E*: tissue slice of the region containing the carotid chemoreceptors and nodose ganglion. During the injection of tracers, Lumafluor Retrobeads were coinjected along with Dextran-conjugated anterograde tracers into the ND (red, *B*) and CB (green, *C*). *D*: brightfield image to demonstrate morphology of the area. *E*: merge image indicating exclusive injection with no spillover (×20 scale bar = 20 μm). *F*: ×10 magnification of the nucleus tractus solitarius (NTS) and ×40 images of each separated channel for CB and ND injections along with merged image (×40, scale bar = 20 μm). *G*: ×10 magnification of the nucleus ambiguus (NA) and ×40 images of each separated channel for CB and ND injections along with merged image (×40 scale bar = 20 μm). Images were taken with Leica Thunder 3 D Culture Imaging system. *H*: the χ^2^ test (df = 7.939, *P* = 0.047) demonstrates a high proportion of co-labeled neuron cell bodies in the NTS and NA. These data were taken from *n* = 4 rats. Table shows the count of cell bodies in each condition. Image created with BioRender and published with permission.

To test whether this efferent augmentation was due to converging synapses in the brainstem we coinjected anterograde tracers into the carotid chemoreceptors and nodose/jugular ganglion. We found a significant number of co-labeled neuronal cell bodies in the NTS (Fig. 2, *F* and *G*), complementing previous reports (6–9). A total of 384 NTS cells from 4 rats were labeled with at least one tracer. Of the 384 cells, 56.5% of these neurons had shared sensory input (Fig. 2, *F* and *H*). We further demonstrate convergence in the NA (Fig. 2*G*), the key motor nucleus for the larynx, trachea, and bronchi (2). We show that from a total of 172 NA neurons from 4 rats, which were labeled by at least one tracer, 59.3% were co-labeled (Fig. 2, *G* and *H*). We are confident that these data show convergence and are not a result of fluorophores bleeding into other channels as we chose tracer dyes with Ex/Em spectra which do not overlap. Furthermore, we took great care to ensure the delivery of these tracers to the nodose/jugular ganglion and carotid bodies discretely and confirmed injection with Retrobeads (Fig. 2, *A*–*E*).

**Figure 3.**
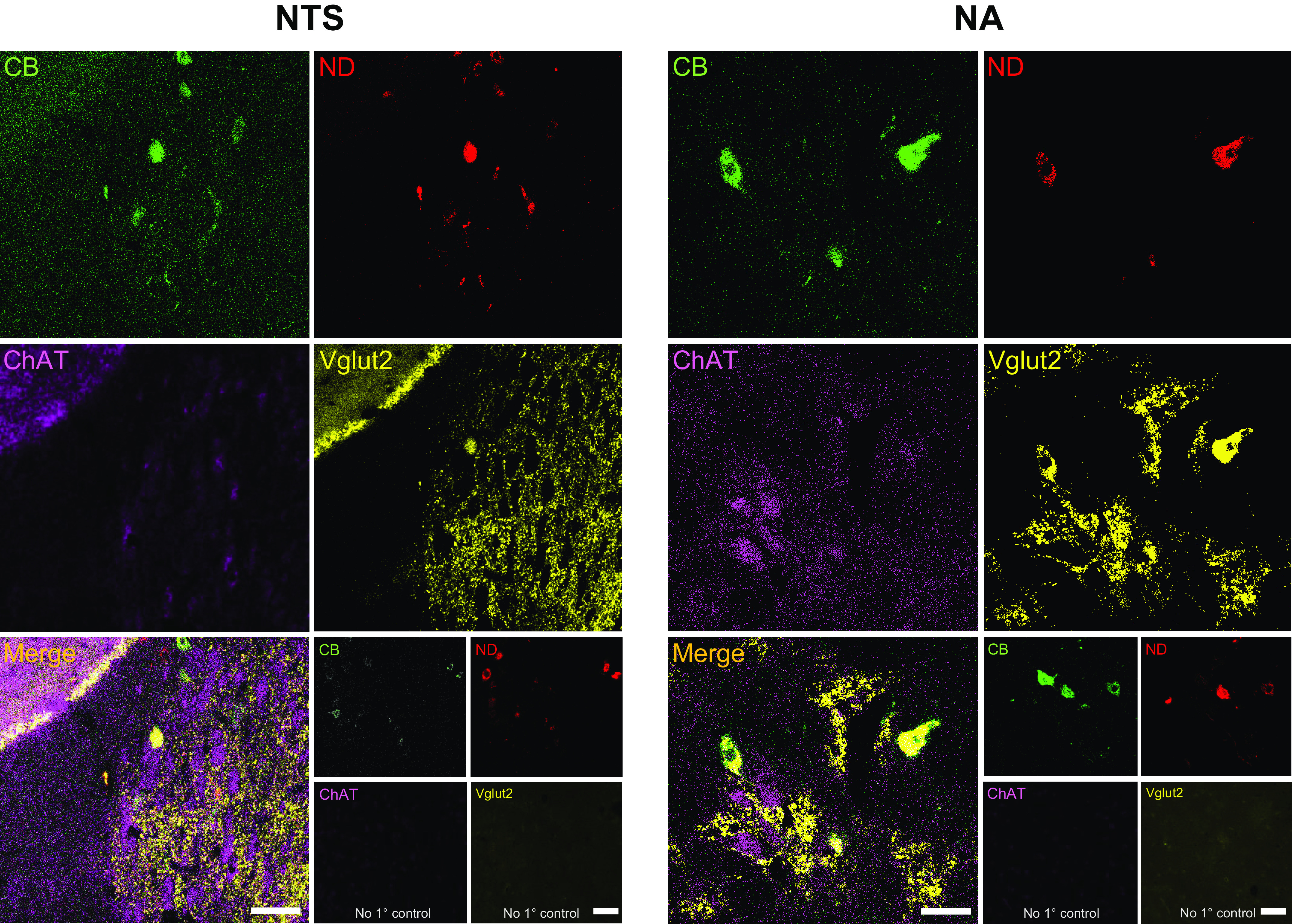
Co-labeled cells express choline acetyltransferase and vesicular glutamate transporter 2. Most neurons labeled with dextran dyes coming from the carotid chemoreceptors (CB, Alexa 488, green) and pulmonary nociceptors (cell bodies in the nodose ganglion, ND, Alexa 647, red) in the nucleus tractus solitarius (NTS) and nucleus ambiguus (NA) were labeled with vesicular glutamate transporter 2 (Vglut2, Cy3, yellow) and in close relation to choline acetyltransferase (ChAT, Dylight 405, magenta) stained cells indicative of an excitatory phenotype. *Bottom right inset* shows a lack of staining but with tracers remaining, when the primary antibodies were omitted to serve as no primary control staining. ×40, scale bar = 20 μm. Images taken with Leica SP8 Confocal Microscope.

To confirm the characteristics of the co-labeled cells, we stained slices with antibodies for ChAT and Vglut2. We show that the NTS is stained with Vglut2 and ChAT with a majority of co-labeled neurons expressing Vglut2 confirming an excitatory phenotype (Fig. 3) (28–30). Cells within the NA were in close vicinity with ChAT-stained cells and co-labeled cells were also positive for Vglut2 in this region (Fig. 3) (28–30). No primary controls demonstrated the presence of labeled cells with dextran tracer dyes but a lack of ChAT or Vglut2 staining (Fig. 3).

## DISCUSSION

We provide neuroanatomical evidence to confirm pulmonary nociceptor and carotid chemoreceptor convergence in the NTS using anterograde tracers. We provide new evidence demonstrating the convergence of pulmonary nociceptors and carotid chemoreceptors in the NA. We show that this convergence results in >50% increase in vagal efferent activity when pulmonary nociceptors and carotid chemoreceptors are co-stimulated.

Stimulation of pulmonary nociceptors results in respiratory defensive reflexes such as cough, bronchoconstriction, and apnea (1). Emerging evidence demonstrates that in addition to the multimodal sensory nature of the carotid chemoreceptors, stimulation can also result in multireflex outcomes (31), such as defensive pulmonary reflexes (14, 19–22). Therefore, the ability of nonpulmonary afferents to elicit pulmonary reflexes points to a degree of overlapping inputs in sensory and efferent brainstem nuclei.

Previously, Paton (7) recorded from single NTS neurons while singly stimulating cardiorespiratory sensory afferents from multiple anatomical locations in the WHBP (8) building on previous data which demonstrated NTS sensory convergence in vivo (6, 9). However, in vivo or singly perfused in situ preparations do not allow precise stimulus delivery as bolus injection or prolonged infusions may circulate to other sensory afferents. Moreover, none of these investigations attempted to characterize dual stimulus delivery to multiple sensory afferents simultaneously nor did they investigate how this would affect the resulting motor output directed to the airways. Our experiments are among the first to precisely control stimulus delivery to pulmonary nociceptors, carotid chemoreceptors, and the brainstem. Our preparation allows precise control of stimuli to the carotid chemoreceptors and brainstem by separately perfusing each with media drawn from separate tonometers (11, 12), which allows prolonged stimulation with natural stimuli like hypoxia instead of the exogenous application of NaCN (7, 8). Our preparation used a further modification to maintain an intact pulmonary circuit so that pulmonary nociceptors could be stimulated (Fig. 1*A*). The vagus neurogram demonstrates typical respiratory activity and captures all traffic which would be directed toward the trachea and bronchi. By injecting ATP (23–25) and fungal protease (26, 27) into the lungs, we demonstrate excitation of efferent vagal output, which coincides with a phrenic apneic phase (Fig. 1*B*). These parameters were augmented when hypoxia was delivered to the carotid chemoreceptors. We also suppressed brainstem chemoreceptors as the brainstem and carotid chemoreceptors compete to control phrenic activity, i.e., suppression of one relinquishes full efferent control of the other (13); therefore, hypocapnic suppression of the brainstem reveals full carotid chemoreceptor excitation. Our data demonstrate that carotid chemoreceptors and pulmonary nociceptors synergize defensive airway reflexes without brainstem chemoreceptor input.

In accord with previous investigations of sensory convergence, we too saw ∼50% convergence between the carotid chemoreceptors and pulmonary nociceptors (7). Although electrophysiological properties are afforded by directly recording from NTS neurons when delivering stimuli to afferents (6–9), these methods precluded sampling of a large number of neurons. The present data extend previous data by showing the convergence of pulmonary vagal sensory afferents and carotid bodies in the NTS and by showing additional convergence in the NA. Previous retro/anterograde tracing studies demonstrated that carotid chemoreceptors and vagal afferents do indeed synapse onto the NTS and NA (2, 5, 32). However, our study is among the first to demonstrate anatomical convergence of both sets of afferents simultaneously in both nuclei. Glutamatergic and cholinergic markers were expressed in the region of co-labeled cells in the NTS and NA (cholinergic - ChAT and glutamatergic - Vglut2) confirming common markers found in these regions (28–30) and an excitatory phenotype of dextran-labeled cells (Fig. 3). Although we stained for excitatory markers, the circuitry of both regions also express inhibitory markers such as GABA (33, 34).

The precise pathway which resulted in an augmentation of vagal and phrenic efferent activity is complex. Given that both afferent sets innervated the NA, it is likely that a shared stimulation of these neurons resulted in heightened vagal efferent activity which would increase the stimulus to target organs and enhance the reflex outcome. Furthermore, we suggest that the augmented vagal activity resulting from sensory convergence results in enhanced airway defensive reflexes. The rise in phrenic motor output is more complex as respiratory rhythm and burst characteristics are governed by a complex respiratory network (2–4). A further investigation into reflex modulation by afferent sensory convergence is warranted as it is possible that other reflexes could be increased or decreased.

Given that we injected the nodose/jugular ganglion, we also expected to see tracers present in the Pa5 region. However, a limitation of our study may be that we did not see much tracer labeling, if any, in the Pa5 region which is innervated mostly by the jugular ganglion (1, 2, 32). Because of the rostral-caudal orientation of the jugular-nodose ganglion, it is likely that injection of the tracers to the vagal ganglia was predominantly delivered to the nodose ganglion as the jugular ganglion lies deeper in the skull base and precludes direct injection (35). Our interest was to delineate pulmonary nociceptor and carotid chemoreceptor convergence. Given that the nodose ganglion houses the predominance of nociceptors, our tracer data are consistent with assessments herein. The data in this study complement a previous report which used genetic methods to identify lung sensory neuron subsets in the NTS and Pa5 regions (36). We note that our study is only a first step to delineating sensory convergence in the brainstem. Future work involving genetic methodologies to parse out specific cell types involved with sensory convergence would assist our understanding of which stimuli and reflexes may be affected in the NTS, NA and, possibly, other nuclei. Furthermore, our vagal neurogram captures motor traffic which is likely directed to the trachea and bronchi. However, our neural recordings cannot delineate the complexity or definitively identify specific motor unit subsets. Strategies which can identify the specificity of vagal efferent traffic (37), such as single-unit recordings, in the context of sensory convergence-mediated augmentation of reflex output would further assist in our understanding of this complex physiology.

In summary, we demonstrate the functional convergence between carotid chemoreceptors and pulmonary nociceptors and give new data to suggest that there may be a direct activation of efferent activity directed to the larynx and bronchi by the carotid chemoreceptors. Our study gives further evidence for multiple pathways and the enhancement of airway defensive reflexes during co-stimulation of these pathways, likely due to convergence within afferent and efferent nuclei.

## ETHICAL APPROVAL

These studies were approved by The Lundquist Institute for Biomedical Innovation at Harbor UCLA Medical Center Animal Care and Use Committee (Assurance Number: D16-00213), in accordance with the Institutional protocol 32183.

## DATA AVAILABILITY

The datasets used and/or analyzed during the current study are available from the corresponding authors on reasonable request.

## GRANTS

This study was supported by NIH National Center for Advancing Translational Science (NCATS) UCLA CTSI Grant No. UL1TR001881a KL2 (UL1TR001881). N.J. is further supported by the Parker B Francis Fellowship.

## DISCLOSURES

No conflicts of interest, financial or otherwise, are declared by the authors.

## AUTHOR CONTRIBUTIONS

N.J. conceived and designed research; J.Z. and N.J. performed experiments; J.Z. and N.J. analyzed data; N.J. interpreted results of experiments; J.Z. and N.J. prepared figures; N.J. drafted manuscript; J.Z. and N.J. edited and revised manuscript; J.Z. and N.J. approved final version of manuscript.
